# Supplementary material for: Photo-thermo semi-hydrogenation of acetylene on Pd1/TiO2 single-atom catalyst
Source: Nat Commun. 2022 May 12;13:2648. doi: 10.1038/s41467-022-30291-x (PMC9098498; doi:10.1038/s41467-022-30291-x)
Supplement: Supplementary file 1 — Supplementary Information [file 41467_2022_30291_MOESM1_ESM.pdf]

Supplementary Information

Photo-thermo semi-hydrogenation of acetylene on Pd_1/TiO_2 single-atom catalyst

Yalin Guo,[1,2,†] Yike Huang,[1,2,†] Bin Zeng,[2,3] Bing Han,[1,2] Mohcin AKRI,[1] Ming Shi,[2,3] Yue Zhao,[3] Qinghe Li,[1] Yang Su,[1] Lin Li,[1] Qike Jiang,[4] Yi-Tao Cui,[5] Lei Li,[6] Rengui Li,[3,*] Botao Qiao,[1,*] and Tao Zhang[1]

[1]CAS Key Laboratory of Science and Technology on Applied Catalysis, Dalian Institute of Chemical Physics, Chinese Academy of Sciences, Dalian 116023, China

[2]University of Chinese Academy of Sciences, Beijing 100049, China

[3]State Key Laboratory of Catalysis, Dalian National Laboratory for Clean Energy, Dalian Institute of Chemical Physics, Chinese Academy of Sciences, Dalian 116023, China

[4]Dalian National Laboratory for Clean Energy, Dalian Institute of Chemical Physics, Chinese Academy of Sciences, Dalian 116023, China

[5]SANKA High Technology Co. Ltd. 90-1, Kurimachi, Shingu-machi, Tatsuno, Hyogo, 679-5155, Japan

[6]Synchrotron Radiation Research Center, Hyogo Science and Technology Association, Hyogo, 679-5165, Japan

†These authors contributed equally

*Email: bqiao@dicp.ac.cn; rgli@dicp.ac.cn

Supplementary Methods

General methods of computational details

In this study, first principle (Density Functional theory, DFT) calculations are performed by using CP2K-8.1 software package.[3,4] In CP2K, Goedecker–Teter–Hutter (GTH) pseudopotentials are used to describe electrons mainly distribute near nuclei, they can also decrease the number of basis functions should be used to expand orbitals.[5-7] Therefore, Kohn-Sham (KS) orbitals are expanded by planewave and Gaussian type basis functions (DZVP-MOLOPT-SR-GTH) with cutoff value 400 Ry and 40 Ry respectively after convergence test.[8,9] In our case, in KS energy functional, alpha and beta spin electrons are admitted having different spatial orbitals (unrestricted KS), exchange-correlation interaction (XC) is described by Perdew–Burke–Ernzerhof (PBE) functional that contains Generalized Gradient Approximation (GGA) when optimizing structures.[10] When calculating energies and studying electron population (charge density difference and Mayer bond analysis), XC is described by hybrid functional, the Truncated Coulomb interaction-Long Range Corrected version of PBE0 (PBE0-TC-LRC, where PBE0 = 75% PBE + 25% Hartree-Fock exchange, HFX) functional.[11,12] To accelerate HFX calculation, Auxiliary Density Matrix Method (ADMM) is used,[13] fitting basis set for H, C, O, Ti, Pd elements are pFIT3, pFIT3, pFIT3, FIT10, cFIT11, respectively. Additional corrections such as surface dipole correction and dispersion correction (Grimme-D3) are employed throughout our calculations.[14] Convergence threshold is set to 1E-6 throughout all our calculations. Mayer bond analysis is performed on Multiwfn-3.8-dev software.[15-17] All structure visualizations are supported by VESTA3 software.[18]

For Ti, it is well-known that its electron distribution will be estimated over-delocalized by classical density functional, and this kind of ill-estimation may bring about deviation to optimization of structures, but which actually depends on the construction method and properties of pseudopotentials used. In our case, we do not find significant deviation from experimental measured structure in primitive cell geometry and parameters optimization task, or any

improvement brought about by DFT + U. Therefore, in structural optimization tasks, wavefunction correction method such as Hubbard parameterized correction, *i.e.*, DFT + U is not used. But in energy calculation and electron population analysis tasks, we directly use hybrid functional (PBE0, widely reported that is suitable for excitation simulation tasks,[19] although quite expensive but it can avoid introducing subjective bias (for example, the selection on value of Hubbard parameter U or $U_{eff} = U - J$) and error (U transferable usage across different system) into calculations. Detailed comparison between structures obtained from simulation and experiments are shown in the following Supplementary Table 2.

Model construction

In our experiments, the support is a mixture of anatase and rutile, which indicates the structures of reaction sites are uncertainty, one single model is not reliable to ensure whether light can promote reaction. Therefore, both phases, three structures in total that mostly reported in literature are considered. For anatase, the most stable crystal plane (101) with O_{2c} (two folded coordinated oxygen atom) exposes on outmost surface is used, and then duplicated in three direction as 1×3×3 ($Pd_1Ti_{36}O_{72}$). Pd anchors at two different stable sites that has been reported, respectively.[20,21] For rutile, the most stable crystal plane (110) with O_{2c} and Ti_{5c} (five folded coordinated titanium atom) expose on outmost surface is used, and then duplicated in three direction as 4×2×3 ($Pd_1Ti_{48}O_{96}$). Pd anchors at the most stable site reported by Li et al.[21,22] For every model, a 15-Angstrom-thickness vacuum is added to avoid interaction between replica along z-axis, Supplementary Fig. 15.

For structural optimization tasks, bottom 6 layers of atoms are fixed in Pd/TiO_2-a101-1 and -2 models, bottom 3 layers of atoms are fixed in Pd/TiO_2-r110 model, all other atoms are allowed to relax. Four convergence thresholds are used in this kind of task: structural change (MAX_DR: 3E-3 Bohr), root mean square (RMS) of geometry change (RMS_DR: 1.5E-3 Bohr), the maximum force component of present structure (MAX_FORCE: 4.5E-4 hartree/Bohr), RMS of force of present structure (RMS_FORCE: 3.0E-4 hartree/Bohr).

Constrained DFT

Basic concepts of CDFT and methods used in our work

Traditionally, for excitation calculation, in generalized DFT framework, Time-Dependent Density Functional Perturbation Theory (TD-DFPT) should be used. However, as what mentioned briefly in main text, because in our case we have more than 150 atoms at most in model, the number of states should be calculated grows exponentially with the excitation energy. When excitation energy approaches 3.0 eV (414.13 nm), there are already more than 5,000 states we should calculate shown as Supplementary Fig. 27. What is worse is that in TD-DFPT method, due to traditional subspace diagonalization algorithm that is used to perform wavefunction optimization, to obtain reliable result, it is empirically required that at least 50% more states should be calculated (> 7500 states), this is extremely expensive and furtherly more than what we can afford.

However, in our case, the support we use is TiO_2, a common photocatalytic material where charge-transfer type excitation always occurs, it is intuitive to study whether its charge-transfer type excitation can cause activation of H-H and/or C-C bonds, improve reactivity of our catalysts, Supplementary Fig. 28. Therefore, we turn to use CDFT to simulate possible charge transfer that may be caused by excitation. CDFT is the method that extends traditional KS energy functional by adding (variational) constraints terms and perform conditional optimization, where additional Lagrangian multipliers $\{\lambda\}$ will be optimized during iteration. In our case, CDFT energy functional should be written as Supplementary Eq. (1):

$$E_{CDFT}[\rho,\ \lambda] = \max_{\lambda} \min_{\rho} \left(E_{KS}[\rho] + \lambda \left[\sum_{i=\uparrow,\downarrow} \int w^i(\boldsymbol{r}) \rho^i(\boldsymbol{r}) d\boldsymbol{r} - N_e \right] \right) \tag{1}$$

, where $E_{CDFT}[\rho,\ \lambda]$ has been defined in spin-polarized form, $E_{KS}[\rho]$ is KS energy functional, λ is Lagrangian multiplier of constraint, N_e is the number of electrons we need to constrain, and spin-polarized weight factor $w^i(\boldsymbol{r})$ is defined as Supplementary Eq. (2):

$$w^{i}(\boldsymbol{r})=\frac{\sum_{j\in C}c_{j}P_{j}(\boldsymbol{r})}{\sum_{j\in N}P_{j}(\boldsymbol{r})} \quad (2)$$

, c_j is atomic coefficient that set manually, in our case we set to 1 for each atom. $P_j(\boldsymbol{r})$ is the electronic partitioning-relevant parameter, in our case, it is defined in the same way as Hirshfeld charge. C is collection of atoms need to constrain, N is collection of all atoms.

In our practical CDFT calculations, two methods are used to designate electron donor and acceptor part, corresponding to two different kinds of excitation modes. In the first method (denoted as Method 1), we divide the whole system into two parts as TiO_2 (photosensitive part, electron donor) and Pd-adsorbate composite (electron acceptor, reaction center), 0.5 e are constraint to transfer from TiO_2 to reaction center part; In the second method (Method 2), system is divided into two parts as catalyst (electron donor) and adsorbate (electron acceptor which is the main part obtain electrons and to be activated), 1.0 e are constraint to transfer from catalyst to adsorbate. Energy differences between ground state and constraint charge transfer states are calculated and converted to wavelength unit (nm).

Further CDFT calculations to exclude activation of H_2 and another example of filling orbitals of electrons transferred

As we have shown in Table 1 in the main text, there are some modes left un-certained. Based on results shown in Table 1, it is not enough to draw conclusion whether these modes are energy-favored and if they can show activation effect on chemical bonds of interest, reasons are: (1) If present mode shows wavelength longer than the minimal wavelength (320 nm) of our lamp and does not show activation of chemical bonds, it is still possible for it to show activation effect with a shorter wavelength (i.e., more electrons transferred) that is still longer than minimal wavelength of our lamp. (2) The modes that can be directly judged as possible or impossible are: *(possible)* Already show activation with wavelengths longer than 320 nm, *(impossible)* Do not show activation with wavelengths shorter than 320 nm.

We have particular interests to ensure if our models can show agreement with our finding in

experiments that H_2 activation is not possible or not significant, therefore, we increased number of electrons transferred in Method 2 in Pd/TiO_2-a101-1-H_2*, Pd/TiO_2-a101-2-H_2* and Pd/TiO_2-r110-H_2* models from 1.0 to 1.25, re-calculate bond orders and energy differences (convert to wavelength unit). It is shown that adsorbed hydrogen will not be activated even when being irradiated with 286.78, 285.00 and 241.22 nm ultra-violet light. We also note that wavelengths of mode Pd/TiO_2-r110-C_2H_2*-2H* are significantly large, which may imply it is possible to activate C-C bond with shorter wavelengths. Thus, we increase the number of electrons transferred to check if activation will happen. For Method 1, activation indeed happens starting from wavelength decreases to certain value larger than 661.10 nm, for Method 2, activation happens, see the Supplementary Fig. 33.

Convergence test on grid size used to calculate overlap matrix in Mayer bond order analysis: see the Supplementary Fig. 34.

Supplementary Figures

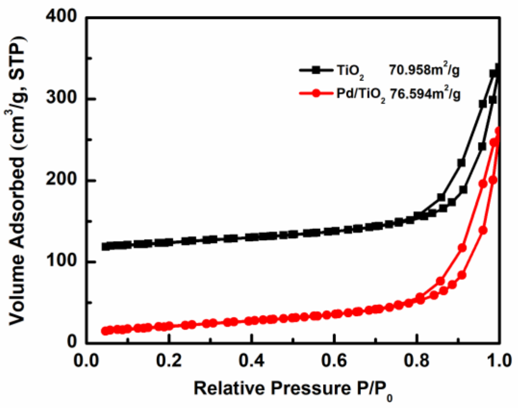

Supplementary Fig. 1 BET Surface Area of TiO_2 and Pd/TiO_2. The N_2 adsorption/desorption isotherm of the TiO_2 and Pd/TiO_2 synthesized by ball milling obtained at 77 K. The samples were degassed at 100 °C for 1 h and followed 300 °C for 4 h before nitrogen adsorption.

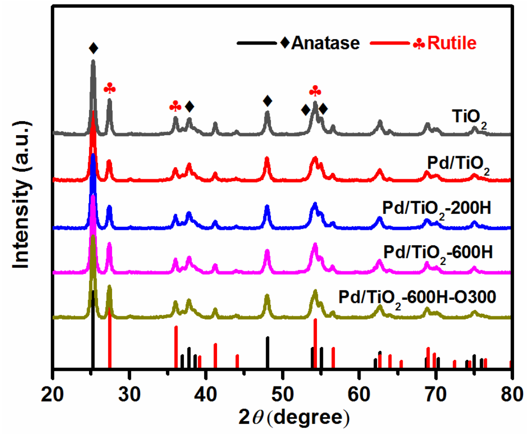

Supplementary Fig. 2 XRD spectra of TiO_2 and Pd/TiO_2 serial catalysts. XRD patterns of TiO_2 and Pd/TiO_2, Pd/TiO_2-200H, Pd/TiO_2-600H, Pd/TiO_2-600H-O300.

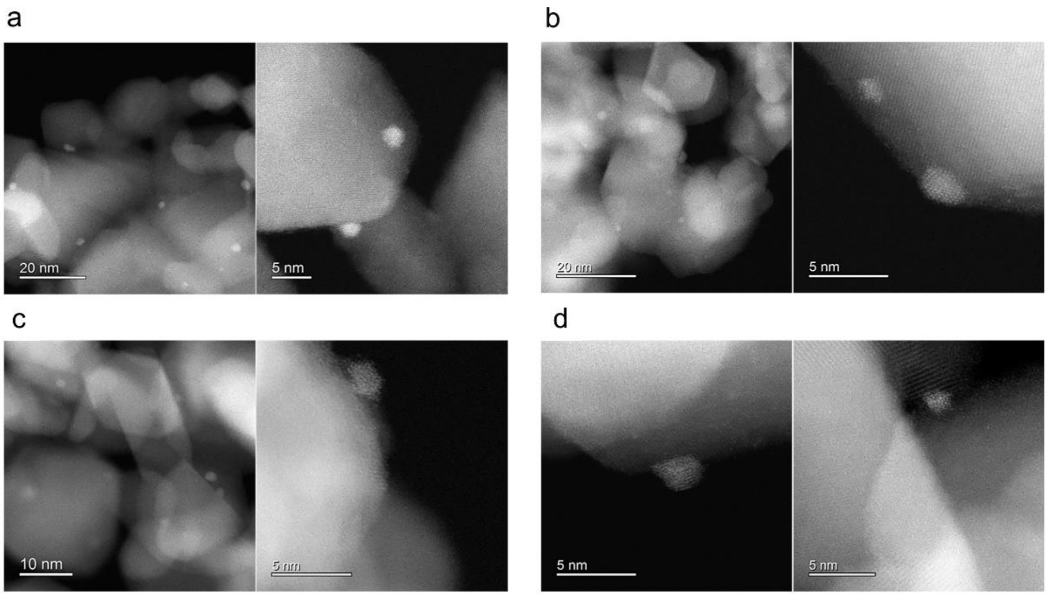

Supplementary Fig. 3 HAADF-STEM characterization of Pd/TiO_2 serial catalysts. HAADF-STEM images of (**a**) Pd/TiO_2, (**b**) Pd/TiO_2-200H, (**c**) Pd/TiO_2-600H, and (**d**) Pd/TiO_2-600H-O300.

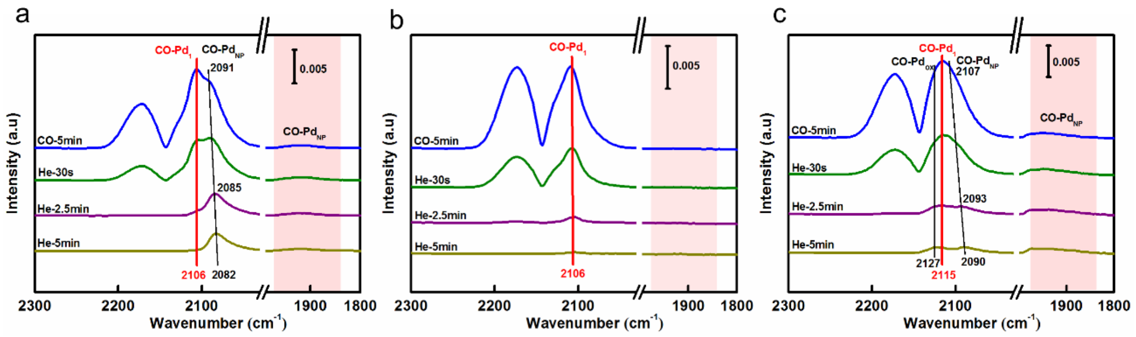

Supplementary Fig. 4 CO-DRIFTS of Pd/TiO_2 serial catalysts. DRIFTS of CO adsorption at saturation coverage and followed by He flush at room temperature on (**a**) Pd/TiO_2-200H, (**b**) Pd/TiO_2-600H, and (**c**) Pd/TiO_2-600H-O300.

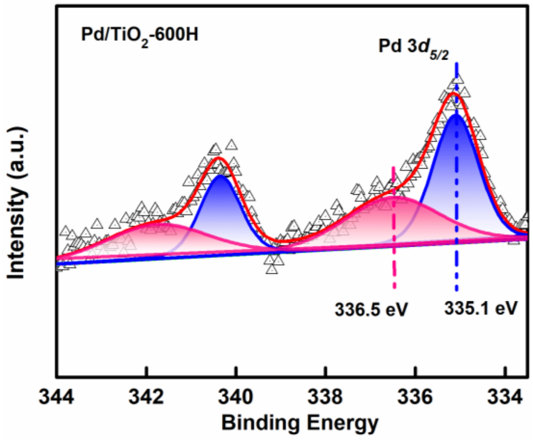

Supplementary Fig. 5 XPS spectrum of Pd/TiO$_2$-600H. Pd 3*d* XPS of Pd/TiO$_2$-600H.

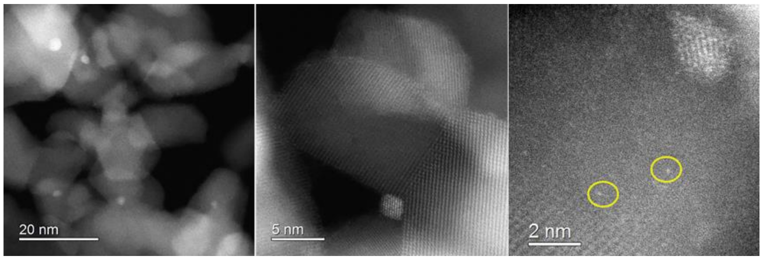

Supplementary Fig. 6 HAADF-STEM characterization of Pd/TiO_2-600H after reaction. HAADF-STEM images of Pd/TiO_2-600H after durability test at 120 ºC for 40 h. Pd single atoms are highlighted in yellow circles.

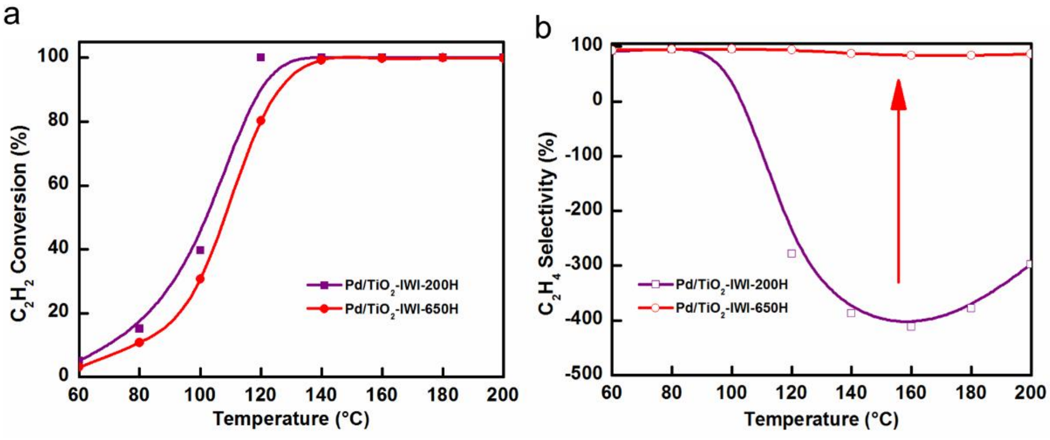

Supplementary Fig. 7 Catalytic Performance of Pd/TiO2-IWI serial catalysts. (**a**) Acetylene conversion as a function of temperature for acetylene semi-hydrogenation, and (**b**) ethylene selectivity over Pd/TiO2-IWI-200H and Pd/TiO2-IWI-650H. Reaction conditions: 1 vol% C_2H_2, 10 vol% H_2, 20 vol% C_2H_4 balanced with He; WHSV = 180 000 mL·h^{-1}·g_{cat}^{-1}.

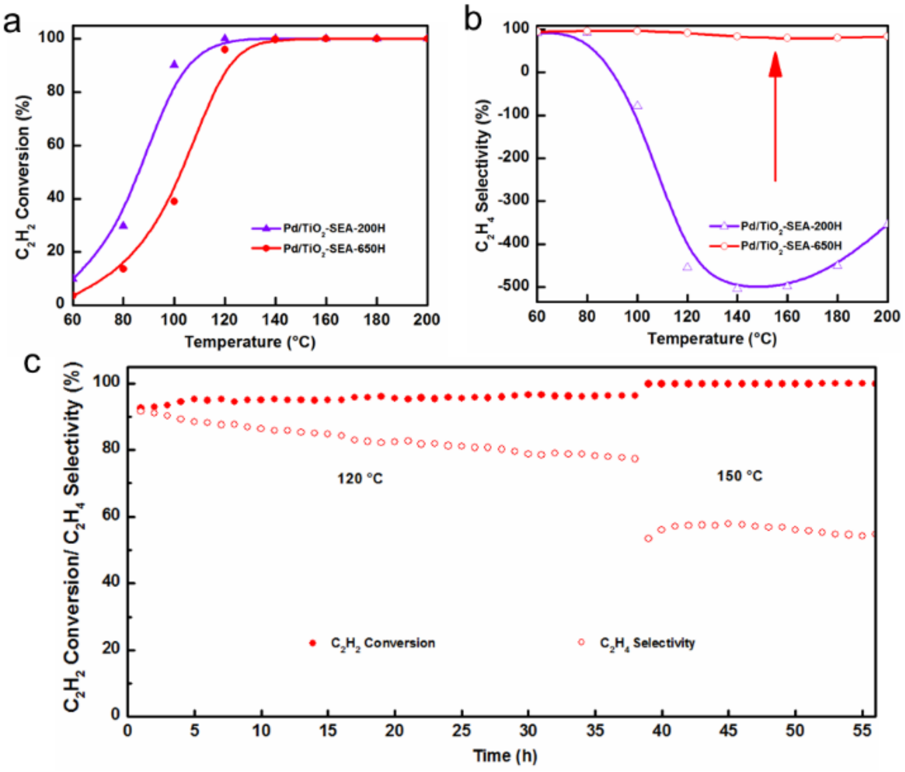

Supplementary Fig. 8 Catalytic Performance of Pd/TiO2-SEA serial catalysts. (**a**) Acetylene conversion and (**b**) ethylene selectivity as a function of temperature for acetylene semi-hydrogenation over Pd/TiO2-SEA-200H and Pd/TiO2-SEA-650H; (**c**) The durability test on Pd/TiO2-SEA-650H at 120 ºC and 150 ºC. Reaction conditions: 1 vol% C_2H_2, 10 vol% H_2, 20 vol% C_2H_4 balanced with He; WHSV = 180 000 $mL \cdot h^{-1} \cdot g_{cat}^{-1}$.

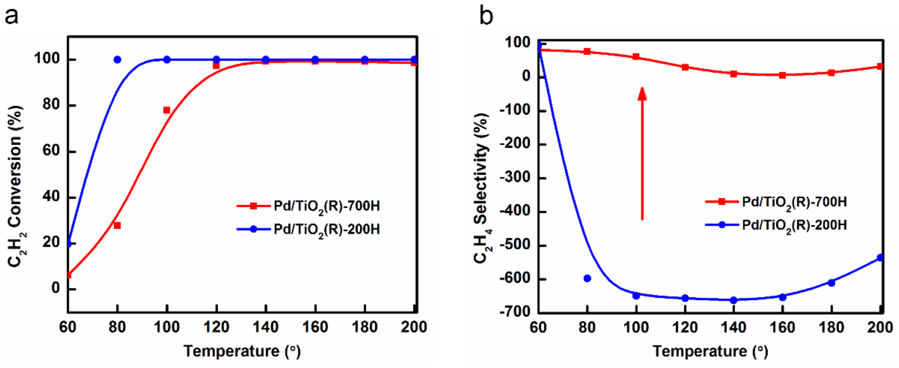

Supplementary Fig. 9 Catalytic Performance of Pd/TiO2(Rutile) serial catalysts. (**a**) Acetylene conversion and (**b**) ethylene selectivity as a function of temperature for acetylene semi-hydrogenation over 0.15 wt% Pd/TiO_2(rutile)-700H and 0.15 wt% Pd/TiO_2(rutile)-200H. Reaction conditions: 1 vol% C_2H_2, 10 vol% H_2, 20 vol% C_2H_4 balanced with He; WHSV = 180 000 mL·h^{-1}·$gcat^{-1}$.

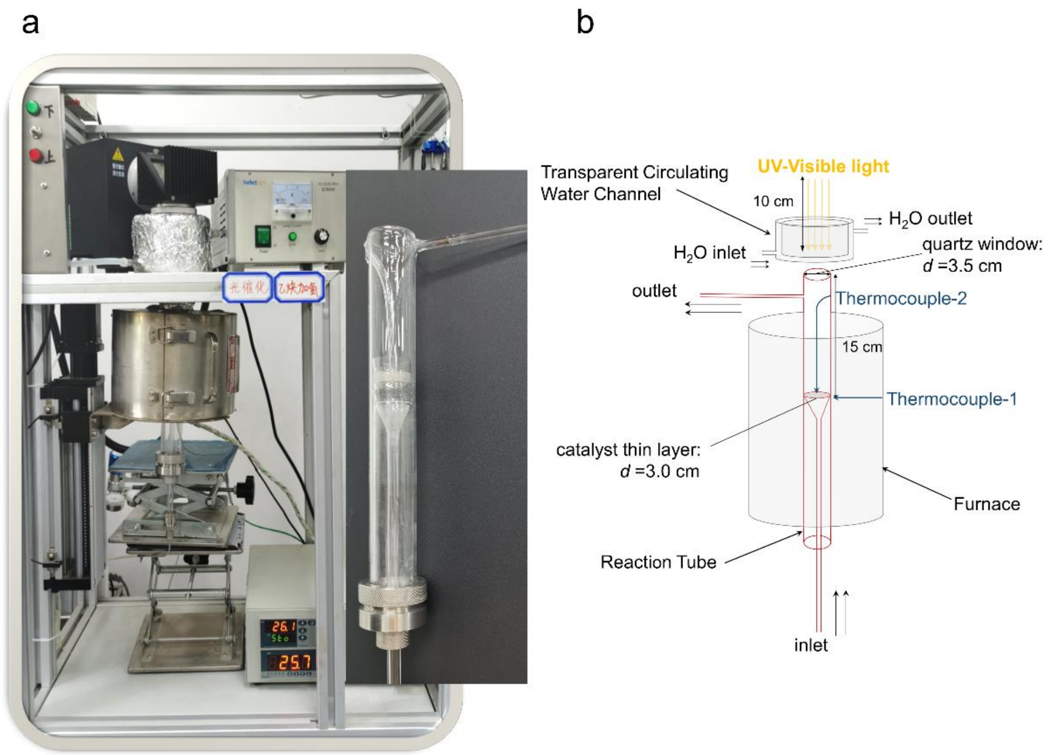

Supplementary Fig. 10 The designed continuous flow fixed-bed reactor. (**a**) The fixed-bed reactor and (**b**) amplification of the furnace part for photo-thermo semi-hydrogenation of acetylene.

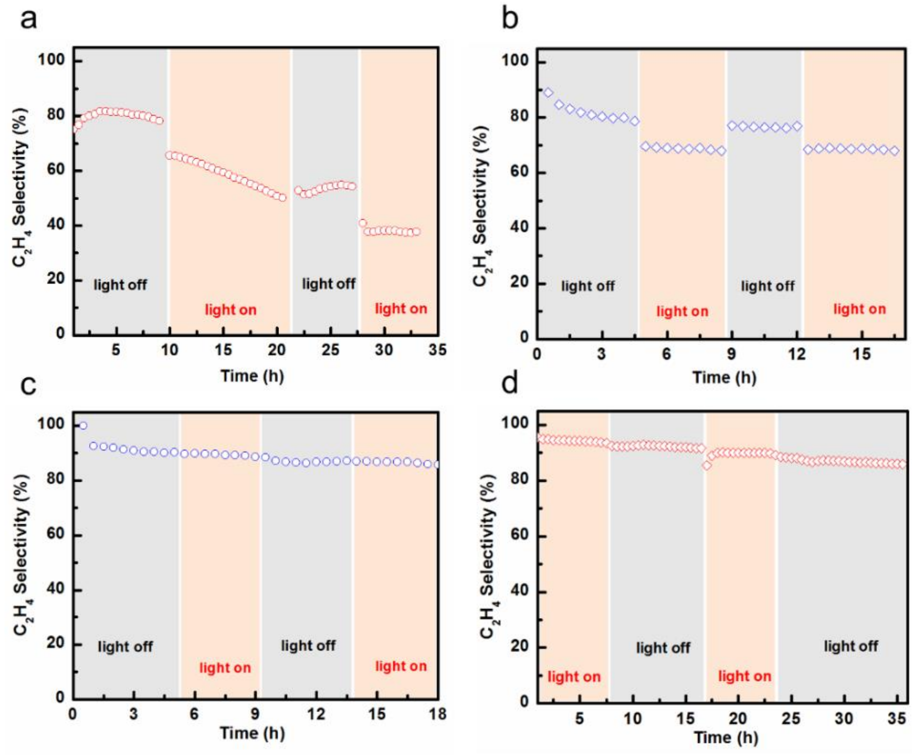

Supplementary Fig. 11 Ethylene selectivity of photo-thermo catalysis over Pd/TiO_2-600H and Pd/Al_2O_3. Ethylene selectivity on the catalyst of 0.15 wt% Pd/TiO_2-600H at 70 ºC in the dark and 60 ºC upon (**a**) full-spectrum light irradiation, power density: 167 mW · cm^{-2}; (**b**) visible light (λ > 420 nm), power density: 141 mW · cm^{-2}; (**c**) λ > 480 nm, power density: 127 mW · cm^{-2}; (**d**) Ethylene selectivity on the catalyst of 0.036 wt% Pd/Al_2O_3 at 70 ºC in the dark and 60 ºC upon full-spectrum light irradiation. Reaction conditions: 1 vol% C_2H_2, 10 vol% H_2, 20 vol% C_2H_4 balanced with He; WHSV = 180 000 mL·h^{-1}·g_{cat}^{-1}.

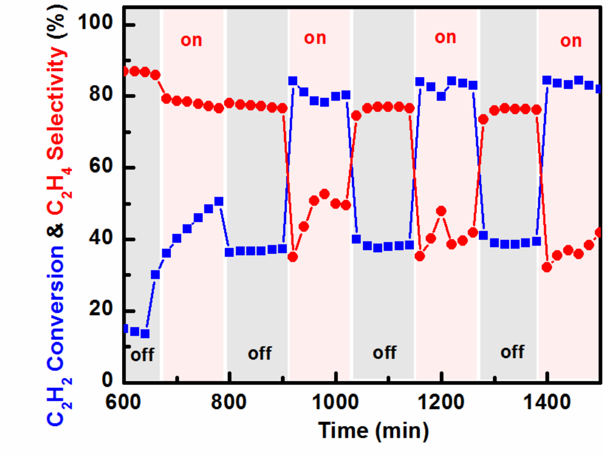

Supplementary Fig. 12 Catalytic Performance of photo-thermo catalysis over Pd/TiO_2-600H for more cycles. Acetylene conversion and ethylene selectivity on the catalyst of 0.15 wt% Pd/TiO_2-600H upon full-spectrum light irradiation, power density: 167 mW · cm^{-2} with 4 cycles.

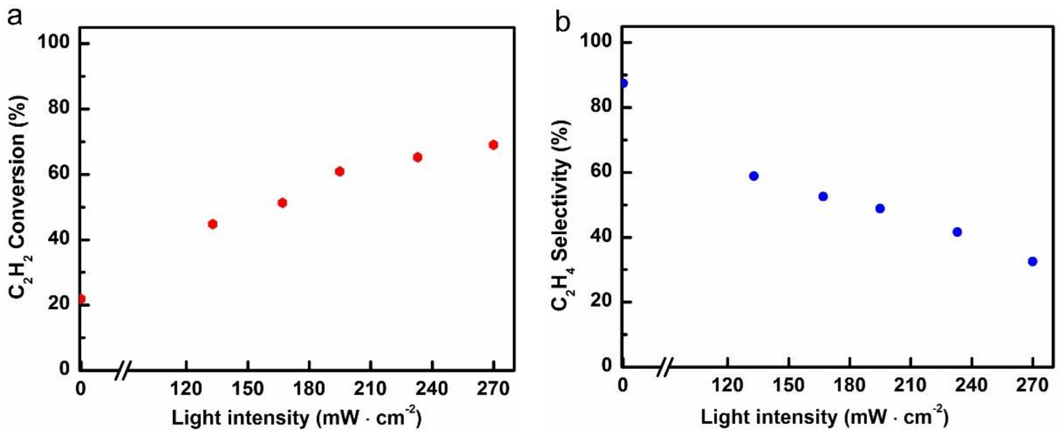

Supplementary Fig. 13 Intensity-dependent experiment upon a full-spectrum light irradiation. (**a**) Acetylene conversion and (**b**) ethylene selectivity as a function of power density over the catalyst of 0.15 wt% Pd/TiO_2-600H at 70 ºC (the test temperature). Reaction conditions: 1 vol% C_2H_2, 10 vol% H_2, 20 vol% C_2H_4 balanced with He; WHSV = 360 000 mL·h^{-1}·g_{cat}^{-1}.

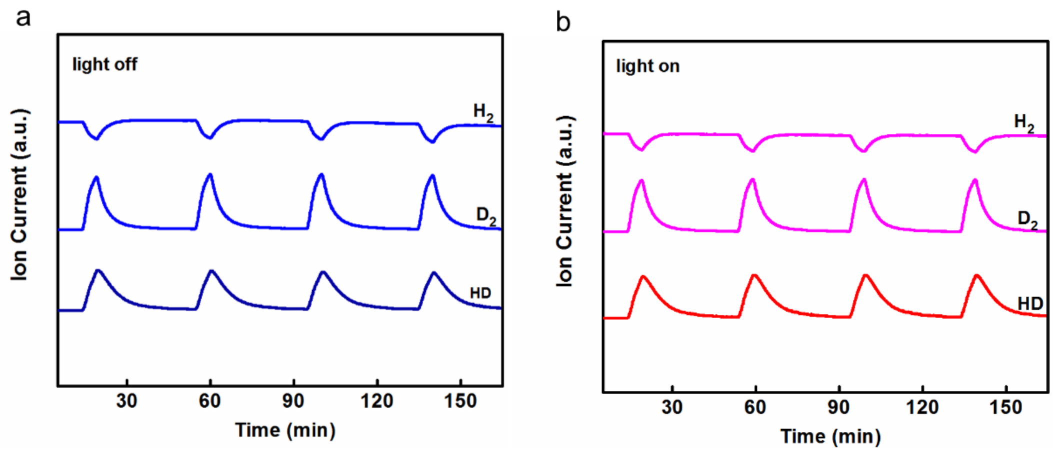

Supplementary Fig. 14 H_2-D_2 isotope exchange reaction over Pd/TiO_2-600H. (**a**) in the dark and (**b**) upon a full-spectrum light irradiation, power density: 167 mW · cm^{-2}. This reaction was performed at 65 °C. Typically, the H_2/He mixture (1: 1) was flowed until stable and then D_2 pulse was sent into the reactor (H_2: He: D_2 = 1: 1: 0.5) until stable, repeating this intake process 4 times. Reaction condition: 30 mL/min, WHSV = 360 000 mL·h^{-1}·g_{cat}^{-1}.

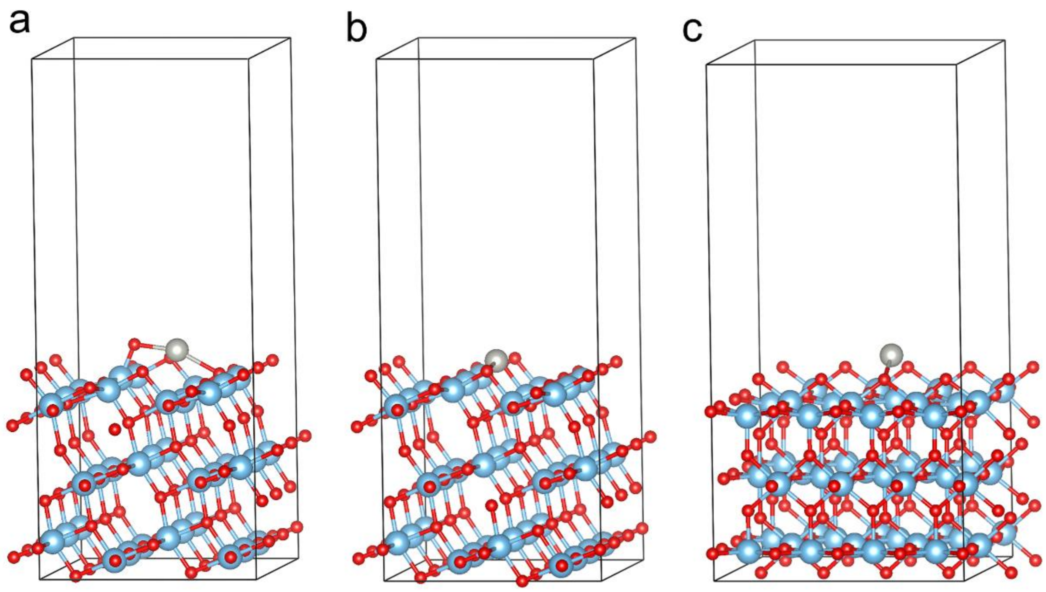

Supplementary Fig. 15 Models construction. (**a**) Pd/TiO_2-a101-1, (**b**) Pd/TiO_2-a101-2 and (**c**) Pd/TiO_2-r110.

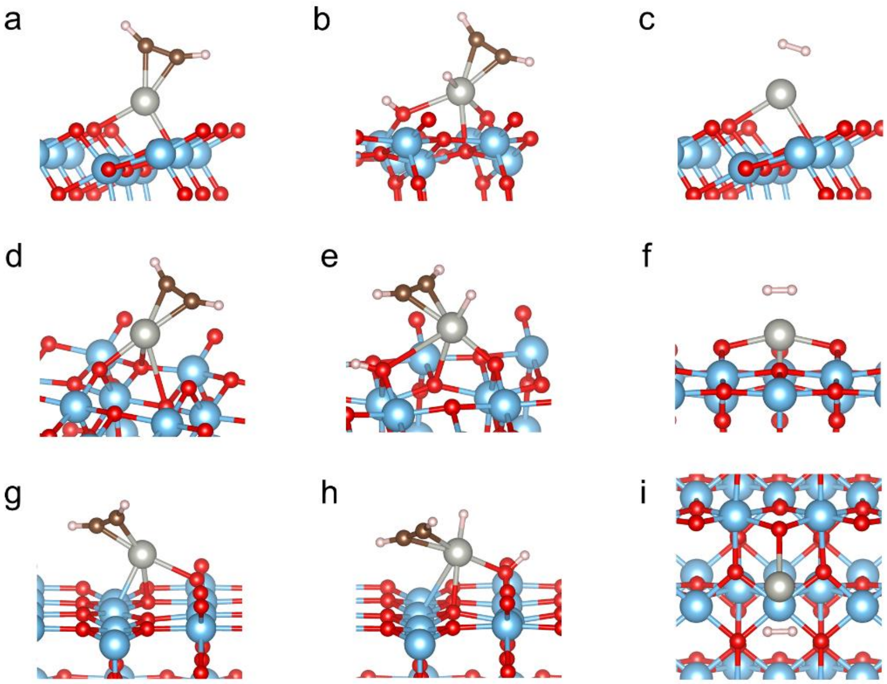

Supplementary Fig. 16 Adsorption structures. (**a**) Pd/TiO_2-a101-1-C_2H_2*, (**b**) Pd/TiO_2-a101-1-C_2H_2*-2H*, (**c**) Pd/TiO_2-a101-1-H_2*, (**d**) Pd/TiO_2-a101-2-C_2H_2*, (**e**) Pd/TiO_2-a101-2-C_2H_2*-2H*, (**f**) Pd/TiO_2-a101-2-H_2*, (**g**) Pd/TiO_2-r110-C_2H_2*, (**h**) Pd/TiO_2-r110-C_2H_2*-2H* and (**i**) Pd/TiO_2-r110-H_2*.

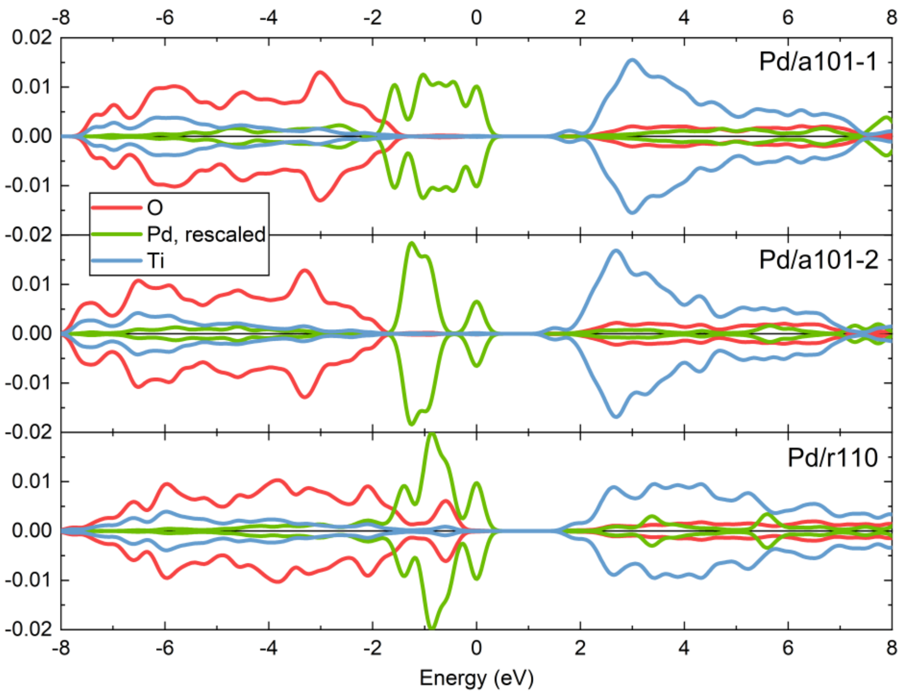

Supplementary Fig. 17 Projected density of states of pristine catalysts. Fermi levels are shifted to 0 eV. Due to there is only one Pd atom in models, to make PDOS of Pd can be clearly seen, PDOS of Pd is rescaled via normalization (PDOS/max(PDOS)) and multiplied by factor 0.02. Significant distribution of states projected on Pd on high energy level indicates high activity towards adsorption. For Pd/a101-1 and Pd/a101-2, bonding orbitals between Pd and support can be found in range -8 ~ -2 eV, corresponding anti-bonding orbitals can be seen in range 2~8 eV. For Pd/r110, additionally at about -0.5 eV, interaction between Pd and (Ti, O) can be seen.

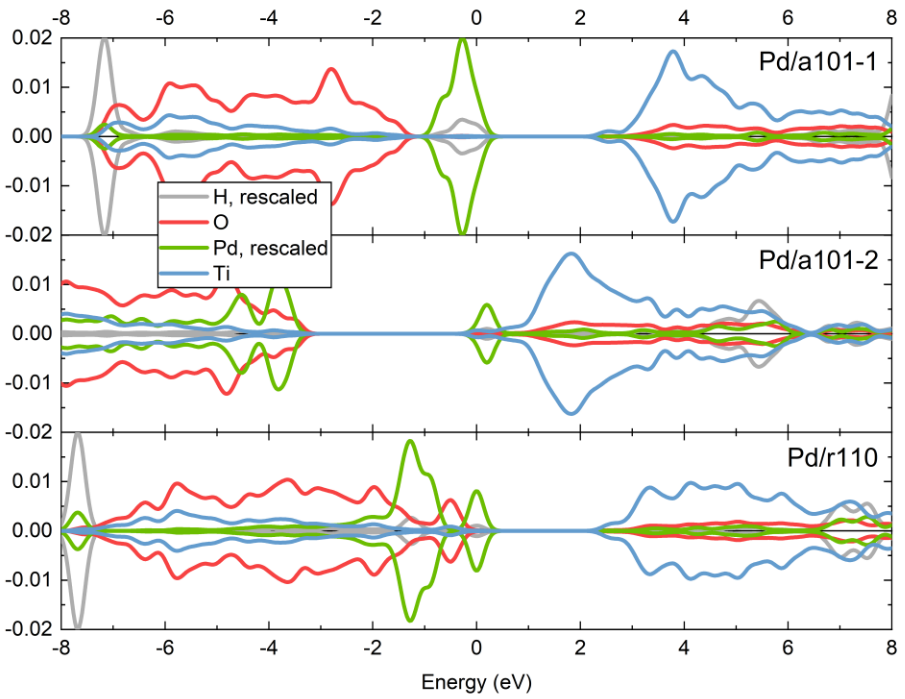

Supplementary Fig. 18 Projected density of states of H_2 adsorbed structures. Fermi levels are shifted to 0 eV. Due to there are only two H atoms and one Pd atom in models, to make PDOS of H and Pd can be clearly seen, PDOS of H and Pd are rescaled via normalization (PDOS/max(PDOS)) and multiplied by factor 0.02. In range -8 ~ 8 eV, significant bonding between Pd and H can be seen in both Pd/a101-1 and Pd/r110 models. On the other hand, gaps between the highest occupied states projected onto Pd or Ti and the lowest unoccupied states of H are approximately larger than 8 eV, corresponding to a wavelength shorter than 155.30 nm.

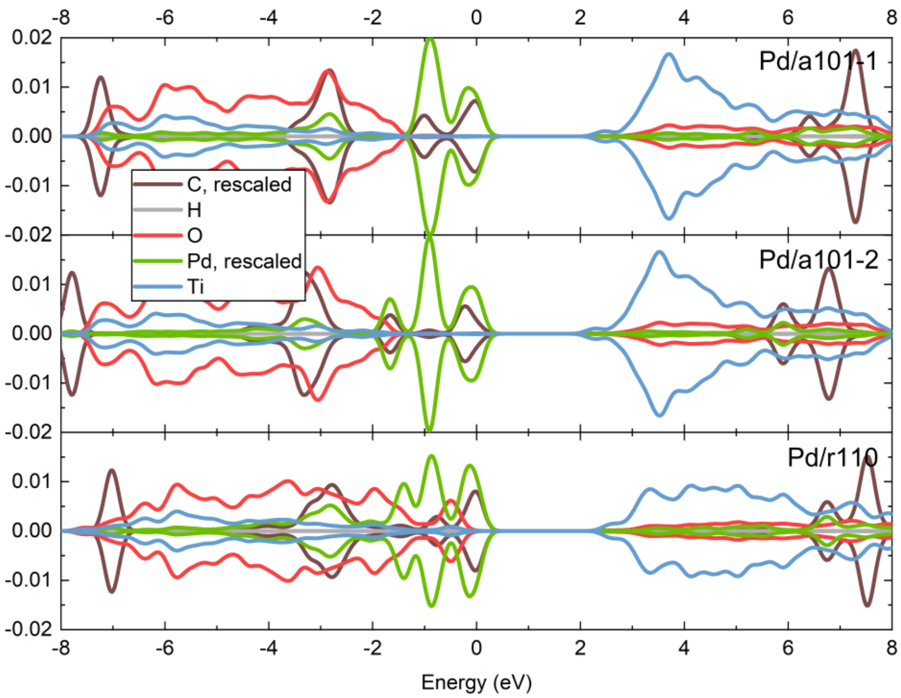

Supplementary Fig. 19 Projected density of states of acetylene adsorbed structures. Fermi levels are shifted to 0 eV. Due to there are only two C atoms and one Pd atom in models, to make PDOS of C and Pd can be clearly seen, PDOS of C and Pd are rescaled via normalization (PDOS/max(PDOS)) and multiplied by factor 0.02. At about 0 eV and -3 eV, mixing of orbitals between Pd and C can be observed. States projected onto Pd in range -2 ~ 0 eV are corresponding well with those in **Supplementary Fig. 17** in nearly the same range. However, gaps between the highest states and lowest unoccupied states projected onto C atoms are quite large, which implies impossibility of C≡C bond activation due to electron excitation in this model.

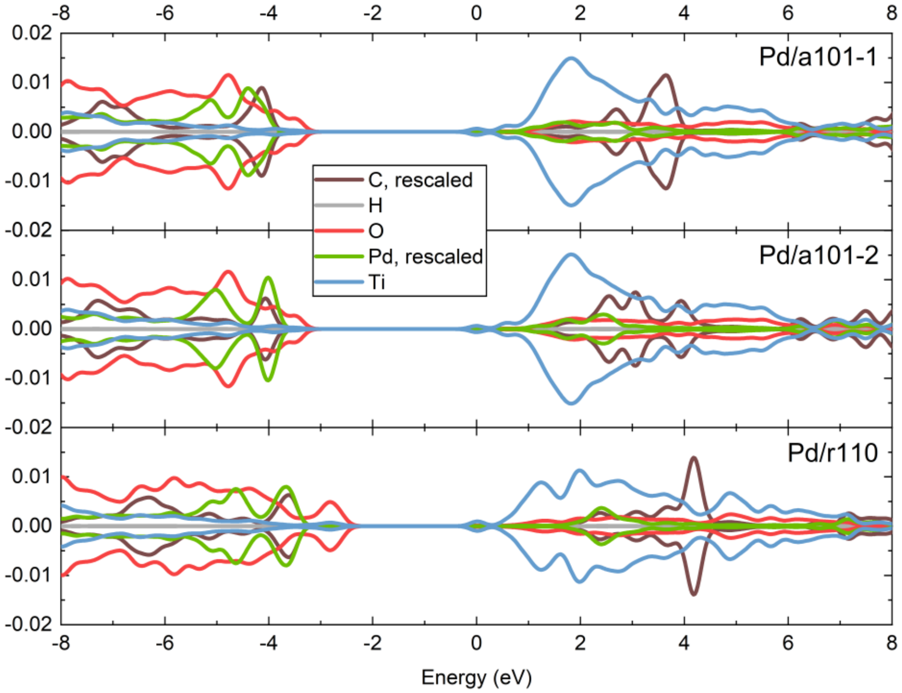

Supplementary Fig. 20 Projected density of states of dissociated hydrogen and acetylene co-adsorbed structures. Fermi levels are shifted to 0 eV. Due to there are only two C atoms and one Pd atom in models, to make PDOS of C and Pd can be clearly seen, PDOS of C and Pd are rescaled via normalization (PDOS/max(PDOS)) and multiplied by factor 0.02. In all these three models, C≡C activation due to electronic excitation is possible because gaps between the highest occupied states projected onto Ti or Pd (for example see **Supplementary Fig. 23**) and the lowest unoccupied states projected onto C (for example see **Supplementary Fig. 24**) are approximately 4 eV, corresponding to a wavelength about 310 nm.

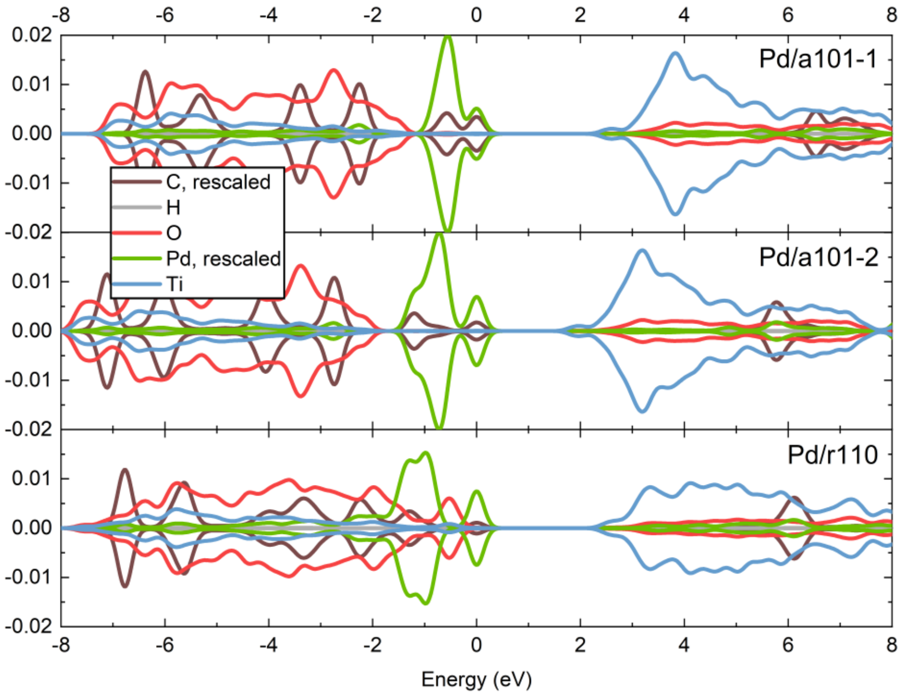

Supplementary Fig. 21 Projected density of states of ethylene adsorbed structures. Fermi levels are shifted to 0 eV. Due to there are only two C atoms and one Pd atom in models, to make PDOS of C and Pd can be clearly seen, PDOS of C and Pd are rescaled via normalization (PDOS/max(PDOS)) and multiplied by factor 0.02. The lowest unoccupied states projected onto C has major contribution of p orbitals corresponding pi bond between C atoms. One can also find that low occupied states projected onto C atoms nearly retain property of isolated molecule, for example, states near -7 eV. However, gap between the highest occupied states projected onto Pd or Ti and pi* orbital at least 6 eV (corresponding wavelength 207 nm) approximately.

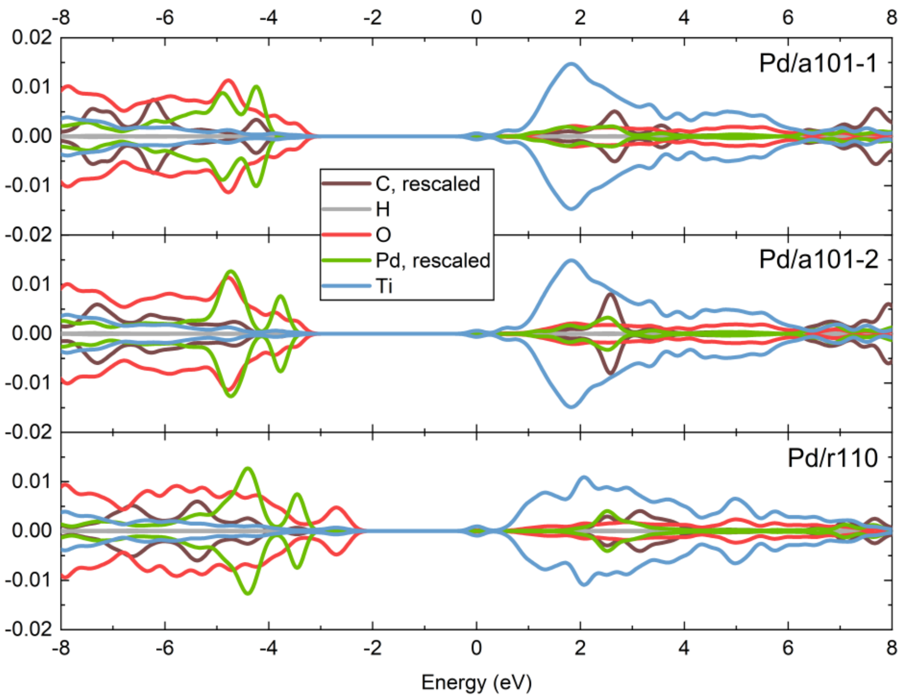

Supplementary Fig. 22 Projected density of states of dissociated hydrogen and ethylene co-adsorbed structures. Fermi levels are shifted to 0 eV. Due to there are only two C atoms and one Pd atom in models, to make PDOS of C and Pd can be clearly seen, PDOS of C and Pd are rescaled via normalization (PDOS/max(PDOS)) and multiplied by factor 0.02. Similar with **Supplementary Fig. 20**, gaps between the highest occupied states projected onto Pd or Ti (for example, see **Supplementary Fig. 25**) and the lowest unoccupied states projected onto C (for example, see **Supplementary Fig. 26**) are 3 eV approximately, corresponding a wavelength 414 nm.

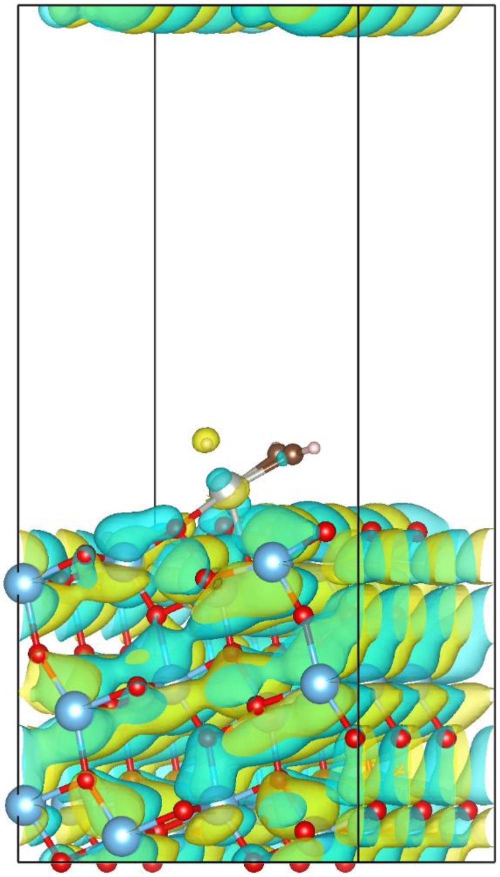

Supplementary Fig. 23 Adsorption structure. The highest occupied state of model a101.Pd-2-ace-2H.

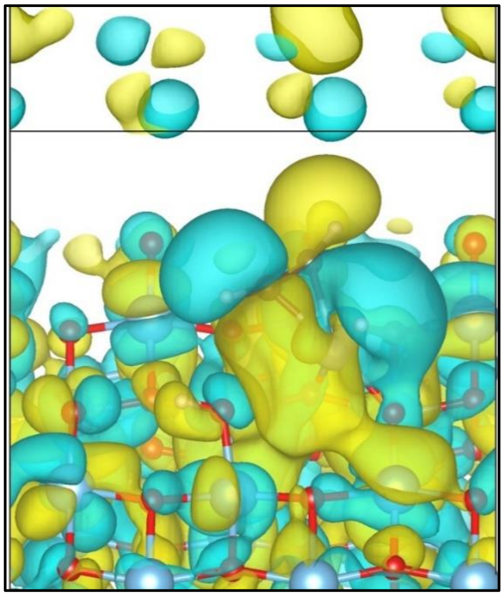

Supplementary Fig. 24 Adsorption structure. The lowest unoccupied states projected onto C atoms in model a101.Pd-2-ace-2H. A feature of pi* orbital symmetry can be seen clearly.

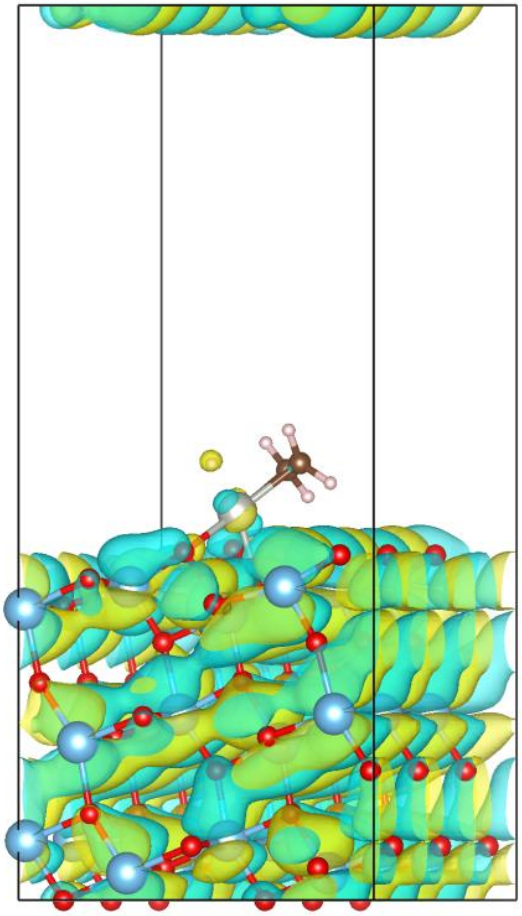

Supplementary Fig. 25 Adsorption structure. The highest occupied state of model a101.Pd-2-eth-2H.

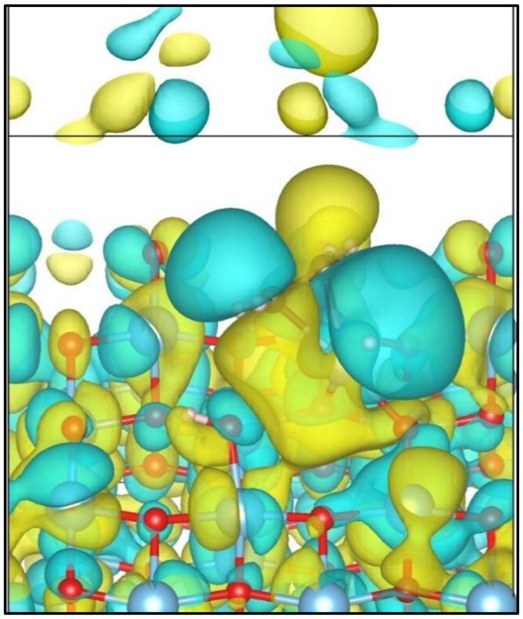

Supplementary Fig. 26 Adsorption structure. The lowest unoccupied states projected onto C atoms in model a101.Pd-2-eth-2H. A feature of pi* orbital symmetry can be seen clearly.

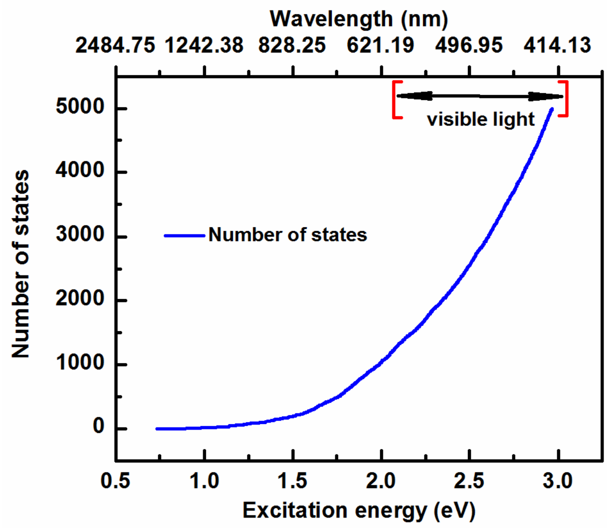

Supplementary Fig. 27 Excitation energy-number of states relationship. Relationship between excitation energy and number of states need to calculate in TD-DFPT run. The range of visible light has been highlighted.

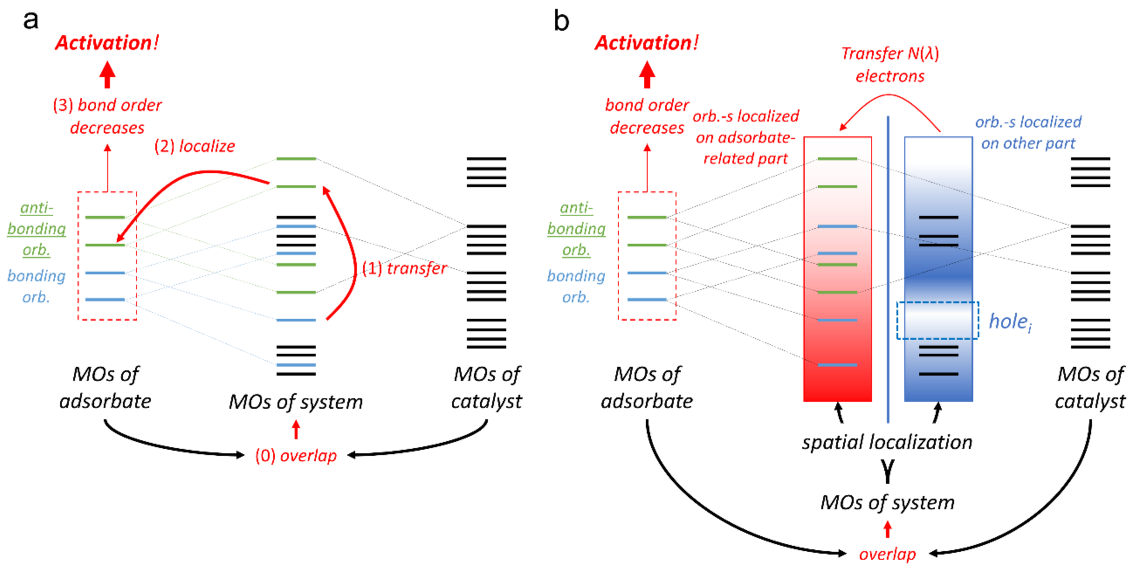

Supplementary Fig. 28 Chemical bond activation process and constrained density functional theory framework. (**a**) Basic picture of chemical bond activation caused by charge-transfer type excitation and (**b**) its approximation picture in constrained density functional theory framework. In our case, electrons are excited to higher energy levels (orbitals), leaving hole state on catalyst spatially. If those orbitals have components of anti-bonding orbitals of adsorbate (H_2 or C_2H_2), decreases in bond order of adsorbates are expected. It is also obvious that the more electrons excited into higher energy levels, the more activation of chemical bonds of adsorbate will occur, and the larger excitation energy (or smaller excitation light wavelength) will be, i.e., in the right figure, N = N(λ), where λ is wavelength of light causing excitation.

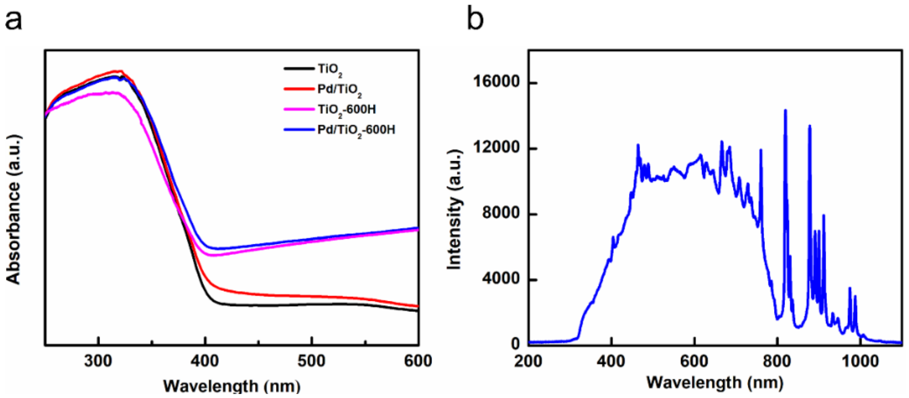

Supplementary Fig. 29 UV-vis spectra and Xe lamp spectrum. (**a**) UV-vis spectra of pure TiO_2, Pd/TiO_2, TiO_2-600H, and Pd/TiO_2-600H; (**b**) spectrum of the Xe lamp used for illumination.

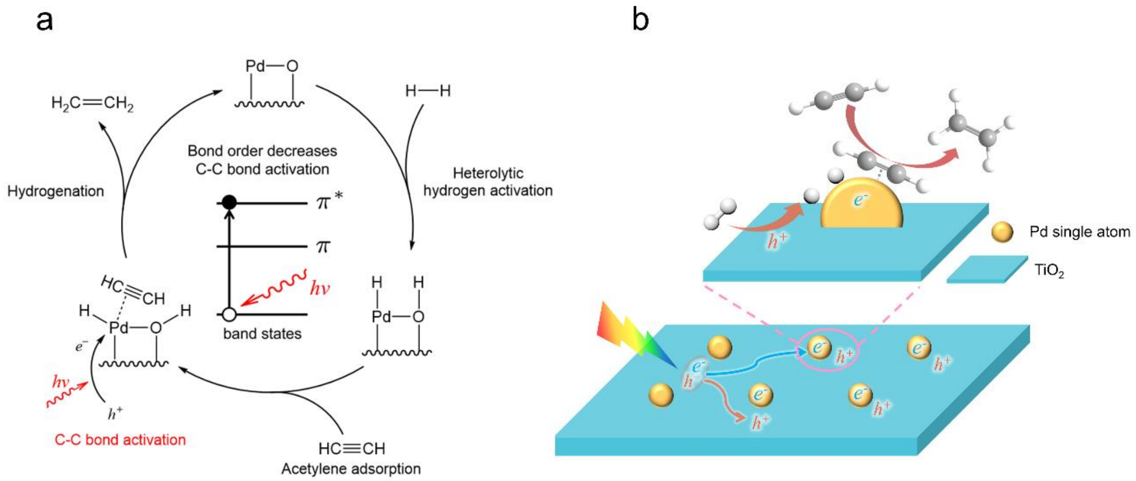

Supplementary Fig. 30 The possible reaction mechanism and process. The detailed (**a**) reaction mechanism and (**b**) reaction process of photo-thermo acetylene semi-hydrogenation over Pd_1/TiO_2 SAC.

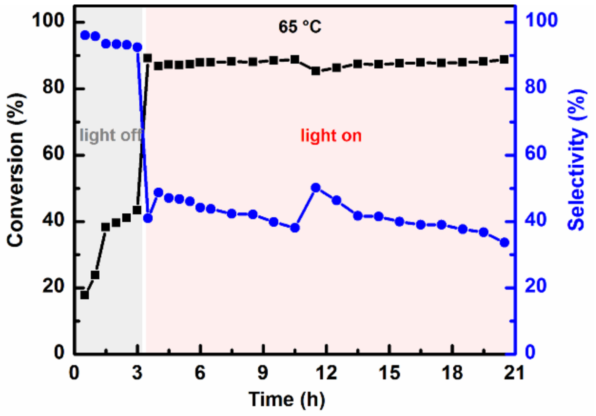

Supplementary Fig. 31 Catalytic Performance of photo-thermo catalysis over Pd/TiO_2 NP-200H. Acetylene conversion and ethylene selectivity over the catalyst of 0.1 wt% Pd/TiO_2 NP-200H at 65 °C in the dark and upon a full-spectrum light irradiation, power density: 167 mW · cm^{-2}, WHSV = 720 000 mL·h^{-1}·g_{cat}^{-1}. Reaction conditions: 1 vol% C_2H_2, 10 vol% H_2, 20 vol% C_2H_4 balanced with He.

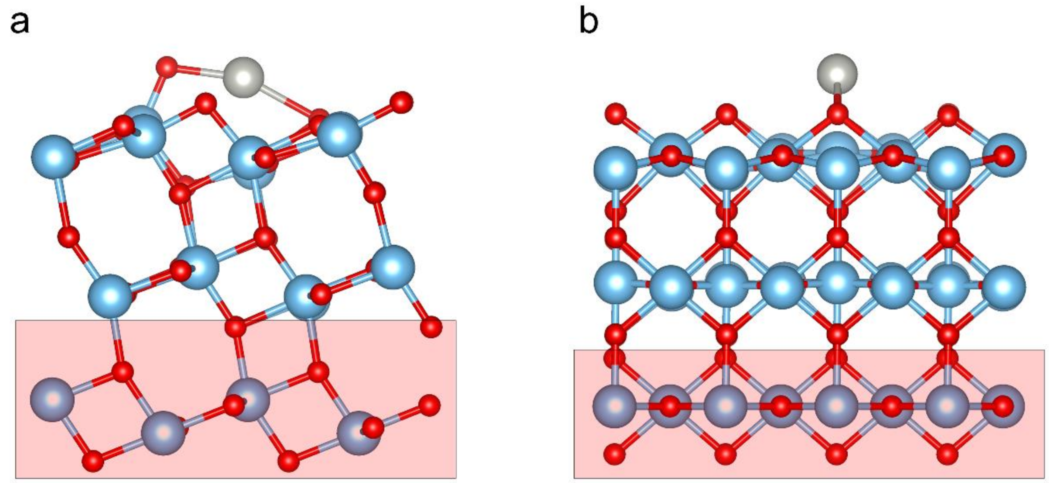

Supplementary Fig. 32 Models construction. Regions where atoms are fixed are indicated with semitransparent red. (**a**) Pd/TiO_2-a101-1 and (**b**) Pd/TiO_2-r110.

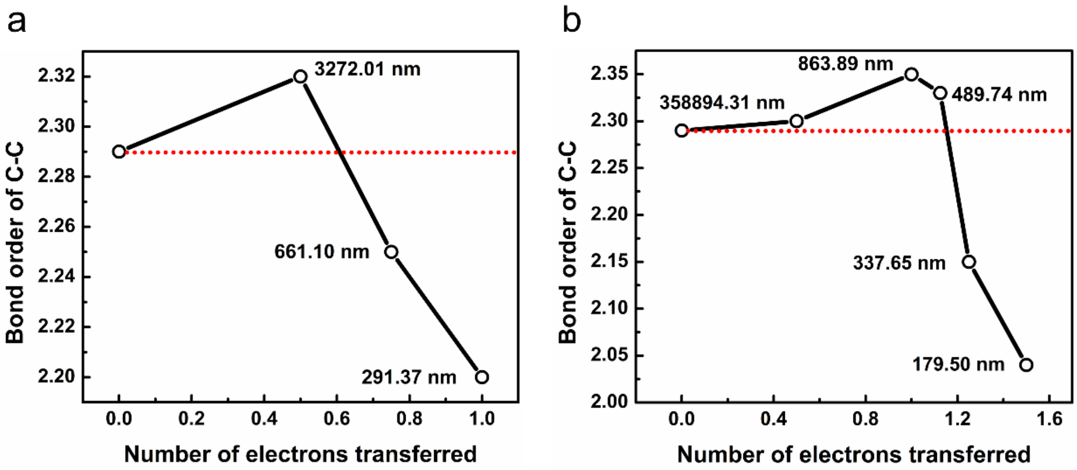

Supplementary Fig. 33 C-C bond activation occurs in Pd/TiO_2-r110-C_2H_2*-2H* system calculated by CDFT. (**a**) Method 1 and (**b**) Method 2. It is noted that wavelengths of mode Pd/TiO_2-r110-C_2H_2*-2H* are significantly large, which may imply it is possible to activate C-C bond with shorter wavelengths. Thus, we increase the number of electrons transferred to check if activation will happen. For Method 1, activation indeed happens starting from wavelength decreases to certain value larger than 661.10 nm, for Method 2, activation happens.

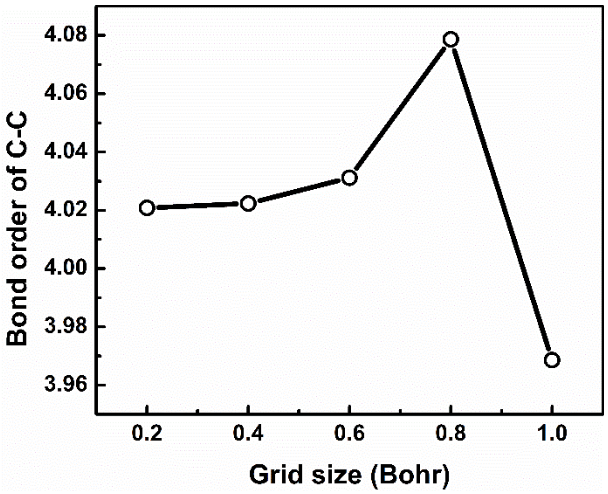

Supplementary Fig. 34 Convergence test. Convergence test on grid size used for Mayer bond analysis implemented in Multiwfn-3.8-dev to calculate overlap matrix. 0.6 Bohr is used in our analysis for the balance between speed and accuracy.

Supplementary Tables

Supplementary Table 1 Catalytic performance of different catalysts in selective hydrogenation of acetylene. (Reaction temperature lower than 200 ^oC)

No.	Catalyst	Reactant Stream (C_2H_2:H_2:C_2H_4)	Temp (°C)	Con (%)	Select (%)	Ref.
**-**	**Pd/TiO_2**	**1:10:20**	**120**	**100**	**>50**	**This work**
**-**	**Pd/TiO_2-UVlight**	**1:10:20**	**60**	**80**	**-**	**This work**
1	$PdAg/Mg_{0.5}Ti_{0.5}O_y$	1:2:100	70	> 99	83.8	[23]
2	$PdIn/MgAl_2O_4$	1:10:100	90	96	92	[24]
3	Pd-Ga/MgO-Al_2O_3	1:2:100	45	85.9	87.2	[25]
4	Pd/MgAl-LDH	1:2:11	70	100	60	[26]
5	$Pd@Ag/TiO_2$	1:2:72	60	82	79	[27]
6	$AgPd/ SiO_2$	1:5:20	65	60	60	[28]
7	Pd–Fe_3O_4–H	1:5:77	80	100	85.1	[29]
8	Pd-[Bmim][Cl]/ Al_2O_3	1:20:100	120	99.5	91	[30]
9	Pd_1/C_3N_4	1:2:50	115	99	83	[31]
10	Pd/COP	1:1.5:82	120	> 99	70-80	[32]
11	Pd_1/N-graphene	1:20:20	125	99	93.5	[33]
12	Mco-PdCu/MgAl-cHTnanoalloy	1:2:100	100	100	82	[34]
13	PdZn/ZnO	1:10:20	110	96	85	[35]
14	Pd SACs@ZIF-8C	1:10:100	120	96	93.4	[36]
15	$BmimBF_4$-0.02Pd_1/HAP	1:10:20	100	>92	>75	[37]

16	$Pd/Ni(OH)_2$	1:8:78	105	80	80	38
17	$Pd\text{-}In/Al_2O_3$	1:36:84	120	82	100	39
18	$Ga_2O_3\text{-}Pd/Al_2O_3$	1:2:110	100	20	95	40
19	PdZn-1.2@ZIF-8C	1:7.7:77	120	85	80	41
20	$CuPd_{0.006}/SiO_2$	1:20:20	160	100	85	42
21	$InPd_2$	1:10:100	200	95	80	43
22	$Fe^{III}\text{-}nZrO_2$	1:167:82	150	90	> 85	44
23	$AgPd_{0.01}/SiO_2$	1:20:20	160	90	80	45
24	$AuPd_{0.025}/SiO_2$	1:20:20	160	44	64	46
25	Op-Au/ SiO_2	1:20:105	175	94	47	47
26	$nFe_2O_3\text{-}nTiO_2$	1:167:82	200	100	90	44
27	$[Fe^{III}\text{-}(H_2O)_6][\ Fe_2^{III}(\mu\text{-}O)_2(H_2O)_6]_{1/2}\{Ni^{II}_4[Cu^{II}_2(Me_3mpba)_2]_3\}\cdot 72H_2O$	1:167:82	150	100	< 90	44
28	$Al_{13}Fe_4$	1:10:100	200	~95	81-84	48
29	$Cu_1/ND@G^a$	1:10:20	200	>95	>98	49
30	$Cu_1/ND@G^b$	1:10:20	180	>99.95	>96	49
31	Na-Ni@CHA	1:16:100	180	100	90	50
32	Ni SAc/N-C	1:20:100	200	90	90	51
33	Ni_3Ga	1:20:100	200	90	77	52
34	$Pd_1/ND@G$	1:10:20	180	100	90	53
35	Pd_3Ga_7	1:10:100	200	99	71	54
36	Pd_2Ga	1:10:100	200	93	76	54
37	Pd_2Ga	1:10:100	200	94	74	55

38	$Pd_2Ga/MgO/MgGa_2O_4$	1:10:100	200	98	70	[55]
39	AuPd-P/TiO_2	1:3:100	200	60	88	[56]
40	$Ni_3Sn_2/MgAl_2O_4$	1:20:100	200	80	80	[52]
41	$Ni_3Ga/MgAl_2O_4$	1:20:100	200	92	77	[52]
42	GaPd-nano	1:10:100	200	>90	65-80	[57]
43	$GaPd_2$-nano	1:10:100	200	~100	60-80	[57]

Supplementary Table 2 Structural characters obtained from simulation and errors respect to experiments.

Structural characters	Optimized (anatase)/ Angstrom	Error respect to experiment/ Angstrom	Optimized (rutile)/ Angstrom	Error respect to experiment/ Angstrom
Ti-O bond 1	1.9817	-0.0588[b)]/-0.0279[c)]	1.9513	+0.0027[d)]/-0.0128[e)]
Ti-O bond 2	1.9349	-0.0609[b)]/-0.0139[c)]	1.9841	+0.0041[d)]/-0.0203[e)]
Cell parameter *a*	3.7901	-0.0601[b)]/-0.0126[c)]	4.5999	+0.0062[d)]/-0.0533[e)]
Cell parameter *b*	3.7901	-0.0601[b)]/-0.0126[c)]	4.5999	+0.0062[d)]/-0.0533[e)]
Cell parameter *c*	9.4894<br>9.4805[a)]	+0.1194[b)]/-0.2584[c)]<br>+0.1105[b)]/-0.2673[c)]	2.9575	-0.0012/-0.0117[e)]

a) Data obtained from DFT + U (ramping), Mulliken method, where $U_{eff,\ Ti}$ = 4.0 eV, $U_{ramping}$ = 0.5 eV. A larger deviation from experimental value indicates accuracy of optimization task will not be better if DFT + U is used.

b) Data resource: 10.1524/zkri.1923.58.1.522, COD ID: 1010942

c) Data resource: https://materialsproject.org/materials/mp-390/

d) Data resource: 10.1107/S010876819100335X, COD ID: 9015662

e) Data resource: https://materialsproject.org/materials/mp-2657/

Supplementary References

1. Li, L. *et al*. SAXS Studies on Agglomerative Silica Suspension under Shear. *AIP* **1027**, 779-781 (2008).
2. Ravel, B. & Newville, M. ATHENA,ARTEMIS,HEPHAESTUS: data analysis for X-ray absorption spectroscopy usingIFEFFIT. *J. Synchrotron Radiat.* **12**, 537-541 (2005).
3. Hutter, J., Iannuzzi, M., Schiffmann, F. & Vandevondele, J. cp2k: atomistic simulations of condensed matter systems. *WIRES. Comput. Mol. Sci.* **4**, 15-25 (2014).
4. Lippert, G., Hutter, J. & Parrinello, M. A hybrid Gaussian and plane wave density functional scheme. *Mol. Phys*. **92**, 477-487 (1997).
5. Krack, M. Pseudopotentials for H to Kr optimized for gradient-corrected exchange-correlation functionals. *Theor. Chem. Acc.* **114**, 145-152 (2005).
6. Goe De Cker, S., Teter, M. & Hutter, J. Separable dual-space Gaussian pseudopotentials. Phys. Rev. *B Condens. Matter.* **54**, 1703-1710 (1995).
7. Hartwigsen, C., Goedecker, S. & Hutter, J. Relativistic separable dual-space Gaussian Pseudopotentials from H to Rn. *Phys. Rev. B* **58**, 3641-3662 (1998).
8. Vandevondele, J. & Hutter, J. Gaussian basis sets for accurate calculations on molecular systems in gas and condensed phases. *J. Chem. Phys.* **127**, 4365-4477 (2007).
9. Vandevondele, J., Krack, M., Mohamed, F., Parrinello, M., Chassaing, T. & Hutter, J. QUICKSTEP: Fast and accurate density functional calculations using a mixed Gaussian and plane waves approach. *Comput. Phys. Commun.* **167**, 103-128 (2005).
10. Perdew, J.P., Burke, K. & Ernzerhof, M. Generalized Gradient Approximation Made Simple. *Phys. Rev. Letters* **77**, 3865-3868 (1998).
11. Guidon, M., Hutter, J.R. & Vandevondele, J. Robust Periodic HartreeFock Exchange for Large-Scale Simulations Using Gaussian Basis Sets. *J. Chem. Theory Comput.* **5**, 3010 (2009).
12. Heyd, J. & Scuseria, G.E. Assessment and validation of a screened Coulomb hybrid density functional. *J. Chem. Phys*. **120**, 7274-7280 (2004).
13. Guidon, M., Hutter, J. & Vandevondele, J. Auxiliary density matrix methods for Hartree-Fock

exchange calculations. *J. Chem. Theory Comput.* **6**, 2348-2364 (2010).

14. Grimme, S., Antony, J., Ehrlich, S. & Krieg, H. A consistent and accurate ab initio parametrization of density functional dispersion correction (DFT-D) for the 94 elements H-Pu. *J. Chem. Phys.* **132**, 154104 (2010).
15. Tian, L. & Chen, F. Multiwfn: A multifunctional wavefunction analyzer. *J. Comput. Chem.* **33**, 580-592 (2012).
16. Mayer, I. Bond orders and valences from ab initio wave functions. *Int. J. Quantum Chem*. **29**, 477-483 (1986).
17. Lu, T. & Chen, Q. Mwfn: A Strict, Concise and Extensible Format for Electronic Wavefunction Storage and Exchange. 10.26434/chemrxiv.11872524 (2020).
18. Momma, K. & Izumi, F. VESTA3 for three-dimensional visualization of crystal, volumetric and morphology data. *J. Appl. Crystallorg.* **44**, 1272-1276 (2011).
19. Adamo, C., Scuseria, G.E. & Barone, V. Accurate excitation energies from time-dependent density functional theory: Assessing the PBE0 model. *Chem. Phys. Letters* **111**, 2889-2899 (1999).
20. Chen, Y. *et al*. Discovering Partially Charged Single-Atom Pt for Enhanced Anti-Markovnikov Alkene Hydrosilylation. *J. Am. Chem. Soc.* **140**, 7407-7410 (2018).
21. Wan, Q., Hu, S., Dai, J., Chen, C. & Li, W.X. Influence of Crystal Facet and Phase of Titanium Dioxide on Ostwald Ripening of Supported Pt Nanoparticles from First-Principles Kinetics. *J. Phys. Chem. C* **17**, 11020-11031 (2019).
22. Wan, Q., Hu, S., Dai, J., Chen, C. & Li, W.X. First-Principles Kinetic Study for Ostwald Ripening of Late Transition Metals on TiO_2 (110). *J. Phys. Chem. C* **123**, 1160-1169 (2018).
23. Liu, Y., Zhao, J., He, Y., Feng, J., Wu, T. & Li, D. Highly efficient PdAg catalyst using a reducible Mg-Ti mixed oxide for selective hydrogenation of acetylene: Role of acidic and basic sites. *J. Catal.* **348**, 135-145 (2017).
24. Feng, Q. *et al*. Isolated Single-Atom Pd Sites in Intermetallic Nanostructures: High Catalytic Selectivity for Semihydrogenation of Alkynes. *J. Am. Chem. Soc.* **139**, 7294-7301 (2017).

25. He, Y., Liang, L., Liu, Y., Feng, J., Ma, C. & Li, D. Partial hydrogenation of acetylene using highly stable dispersed bimetallic Pd-Ga/MgO-Al_2O_3 catalyst. *J. Catal*. **309**, 166-173 (2014).

26. Ma, X.-Y., Chai, Y.-Y., Evans, D.G., Li, D.-Q. & Feng, J.-T. Preparation and Selective Acetylene Hydrogenation Catalytic Properties of Supported Pd Catalyst by the in Situ Precipitation-Reduction Method. *J. Phys. Chem. C* **115**, 8693-8701 (2011).

27. Han, Y., Peng, D., Xu, Z., Wan, H., Zheng, S. & Zhu, D. TiO_2 supported Pd@Ag as highly selective catalysts for hydrogenation of acetylene in excess ethylene. *Chem. Commun.* **49**, 8350-8352 (2013).

28. Zhang, Y., Diao, W., Williams, C.T. & Monnier, J.R. Selective hydrogenation of acetylene in excess ethylene using Ag- and Au-Pd/SiO_2 bimetallic catalysts prepared by electroless deposition. *Appl. Catal., A* **469**, 419-426 (2014).

29. Wu, P. *et al*. Harnessing strong metal-support interactions via a reverse route. *Nat. Commun*. **11**, 3042 (2020).

30. Zhang, Q. *et al*. Outstanding catalytic performance in the semi-hydrogenation of acetylene in a front-end process by establishing a "hydrogen deficient" phase. *Chem. Commun.* **55**, 14910-14913 (2019).

31. Huang, X. *et al*. Enhancing both selectivity and coking-resistance of a single-atom Pd_1/C_3N_4 catalyst for acetylene hydrogenation. *Nano Res.* **10**, 1302-1312 (2017).

32. Yun, S., Lee, S., Yook, S., Patel, H.A., Yavuz, C.T. & Choi, M. Cross-Linked “Poisonous” Polymer: Thermochemically Stable Catalyst Support for Tuning Chemoselectivity. *ACS Catal.* **6**, 2435-2442 (2016).

33. Zhou, S. *et al*. Pd Single-Atom Catalysts on Nitrogen-Doped Graphene for the Highly Selective Photothermal Hydrogenation of Acetylene to Ethylene. *Adv. Mater*. **31**, e1900509 (2019).

34. Liu, Y., He, Y., Zhou, D., Feng, J. & Li, D. Catalytic performance of Pd-promoted Cu hydrotalcite-derived catalysts in partial hydrogenation of acetylene: effect of Pd-Cu alloy formation. *Catal. Sci. Technol*. **6**, 3027-3037 (2016).

35. Zhou, H. *et al*. PdZn Intermetallic Nanostructure with Pd-Zn-Pd Ensembles for Highly Active

and Chemoselective Semi-Hydrogenation of Acetylene. *ACS Catal.* **6**, 1054-1061 (2016).

36. Wei, S. *et al*. Direct observation of noble metal nanoparticles transforming to thermally stable single atoms. *Nat. Nanotechnol*. **13**, 856 (2018).

37. Ding, S. *et al*. Electrostatic Stabilization of Single-Atom Catalysts by Ionic Liquids. *Chem* **5**, 3207-3219 (2019).

38. Hu, M. *et al*. 50 ppm of Pd dispersed on $Ni(OH)_2$ nanosheets catalyzing semi-hydrogenation of acetylene with high activity and selectivity. *Nano Res*. **11**, 905-912 (2017).

39. Cao, Y., Sui, Z., Zhu, Y., Zhou, X. & Chen, D. Selective Hydrogenation of Acetylene over Pd-In/Al_2O_3 Catalyst: Promotional Effect of Indium and Composition-Dependent Performance. *ACS Catal*. **7**, 7835-7846 (2017).

40. Ding, L.B. *et al*. Activating Edge Sites on Pd Catalysts for Selective Hydrogenation of Acetylene via Selective Ga_2O_3 Decoration. *ACS Catal*. **6**, 3700-3707 (2016).

41. Hu, M. *et al*. MOF-Confined Sub-2 nm Atomically Ordered Intermetallic PdZn Nanoparticles as High-Performance Catalysts for Selective Hydrogenation of Acetylene. *Adv. Mater.* **30**, e1801878 (2018).

42. Pei, G.X. *et al.* Performance of Cu-Alloyed Pd Single-Atom Catalyst for Semihydrogenation of Acetylene under Simulated Front-End Conditions. *ACS Catal*. **7**, 1491-1500 (2017).

43. Luo, Y., Alarcón Villaseca, S., Friedrich, M., Teschner, D., Knop-Gericke, A. & Armbrüster, M. Addressing electronic effects in the semi-hydrogenation of ethyne by $InPd_2$ and intermetallic Ga-Pd compounds. *J. Catal*. **338**, 265-272 (2016).

44. Tejeda-Serrano, M. *et al*. Isolated Fe(III)-O Sites Catalyze the Hydrogenation of Acetylene in Ethylene Flows under Front-End Industrial Conditions. *J. Am. Chem. Soc.* **140**, 8827-8832 (2018).

45. Pei, G.X. *et al*. Ag Alloyed Pd Single-Atom Catalysts for Efficient Selective Hydrogenation of Acetylene to Ethylene in Excess Ethylene. *ACS Catal*. **5**, 3717-3725 (2015).

46. Pei, G.X. *et al*. Promotional effect of Pd single atoms on Au nanoparticles supported on silica for the selective hydrogenation of acetylene in excess ethylene. *New J. Chem*. **38**, 2043 (2014).

47. Liu, X., Mou, C.-Y., Lee, S., Li, Y., Secrest, J. & Jang, B.W.L. Room temperature O_2 plasma treatment of SiO_2 supported Au catalysts for selective hydrogenation of acetylene in the presence of large excess of ethylene. *J. Catal.* **285**, 152-159 (2012).

48. Armbruster, M. *et al*. $Al_{13}Fe_4$ as a low-cost alternative for palladium in heterogeneous hydrogenation. *Nat. Mater*. **11**, 690-693 (2012).

49. Huang, F. *et al*. Anchoring Cu_1 species over nanodiamond-graphene for semi-hydrogenation of acetylene. *Nat. Commun*. **10**, 4431 (2019).

50. Chai, Y. *et al*. Acetylene-Selective Hydrogenation Catalyzed by Cationic Nickel Confined in Zeolite. *J. Am. Chem. Soc.* **141**, 9920-9927 (2019).

51. Dai, X. *et al*. Single Ni sites distributed on N-doped carbon for selective hydrogenation of acetylene. *Chem. Commun*. **53**, 11568-11571 (2017).

52. Liu, Y. *et al*. Intermetallic Nix My (M = Ga and Sn) Nanocrystals: A Non-precious Metal Catalyst for Semi-Hydrogenation of Alkynes. *Adv. Mater.* **28**, 4747-4754 (2016).

53. Huang, F. *et al*. Atomically Dispersed Pd on Nanodiamond/Graphene Hybrid for Selective Hydrogenation of Acetylene. *J. Am. Chem. Soc*. **140**, 13142-13146 (2018).

54. Armbruster, M., Kovnir, K., Behren, M., Teschner, D., Grin, Y. & Schlogl, R. Pd-Ga Intermetallic Compounds as Highly Selective Semihydrogenation Catalysts. *J. Am. Chem. Soc.* **132**, 14745-14747 (2010).

55. Ota, A. *et al*. Intermetallic Compound Pd_2Ga as a Selective Catalyst for the Semi-Hydrogenation of Acetylene: From Model to High Performance Systems. *J. Phys. Chem. C* **115**, 1368-1374 (2011).

56. Zhang, S., Chen, C.-Y., Jang, B.W.-L. & Zhu, A.-M. Radio-frequency H_2 plasma treatment of $AuPd/TiO_2$ catalyst for selective hydrogenation of acetylene in excess ethylene. *Catal. Today* **256**, 161-169 (2015).

57. Armbrüster, M., Wowsnick, G., Friedrich, M., Heggen, M. & Cardoso-Gil, R. Synthesis and Catalytic Properties of Nanoparticulate Intermetallic Ga-Pd Compounds. *J. Am. Chem. Soc*. **133**, 9112-9118 (2011).
